# Temporal Changes in Post-Infectious Glomerulonephritis in Japan (1976-2009)

**DOI:** 10.1371/journal.pone.0157356

**Published:** 2016-06-10

**Authors:** Joichi Usui, Takashi Tawara-Iida, Kenji Takada, Itaru Ebihara, Atsushi Ueda, Satoshi Iwabuchi, Takashi Ishizu, Tadashi Iitsuka, Katsumi Takemura, Tetsuya Kawamura, Shuzo Kaneko, Kentaro Sakai, Hirayasu Kai, Tomoka Gomibuchi, Michio Nagata, Masaki Kobayashi, Akio Koyama, Machi Suka, Jai Radhakrishnan, Kunihiro Yamagata

**Affiliations:** 1 Department of Nephrology, University of Tsukuba, Tsukuba, Ibaraki, Japan; 2 Pathology, Faculty of Medicine, University of Tsukuba, Tsukuba, Ibaraki, Japan; 3 Department of Nephrology, Tsukuba Gakuen Hospital, Tsukuba, Ibaraki, Japan; 4 Department of Nephrology, Mito Saiseikai General Hospital, Mito, Ibaraki, Japan; 5 Department of Nephrology, Hitachi General Hospital, Hitachi, Ibaraki, Japan; 6 Department of Medicine, Kensei General Hospital, Sakuragawa, Ibaraki, Japan; 7 Department of Nephrology, Tsukuba Central Hospital, Ushiku, Ibaraki, Japan; 8 Department of Nephrology, Ibaraki Seinan Medical Center Hospital, Sakai, Ibaraki, Japan; 9 Takemura Nephrology Clinic, Kanuma, Tochigi, Japan; 10 The School of Public Health, The University of Tokyo, Tokyo, Japan; 11 Department of Nephrology, Tokyo Medical University Ibaraki Medical Center, Ami, Ibaraki, Japan; 12 Ibaraki Prefectural University, Ami, Ibaraki, Japan; 13 Department of Public Health and Environmental Medicine, The Jikei University School of Medicine, Tokyo, Japan; 14 Division of Nephrology, Columbia University Medical Center, New York, New York, United States of America; Osaka University, Graduate School of Medicine, JAPAN

## Abstract

**Background:**

The incidence of post-infectious glomerulonephritis (PIGN) in developed countries has decreased over the last 50 years. Here we identified the trends of the incidence of PIGN in Japan during the past four decades.

**Methods:**

We explored the frequency, clinicopathological findings, and prognosis of PIGN based on 6,369 cases from the Renal Biopsy Database of our institute in the Kanto region of Japan, diagnosed histologically from 1976 to 2009.

**Results:**

The numbers of PIGN cases were 131 (2.1%) in total, and 2.4%, 1.1%, 2.6% and 2.1% identified in the 1970s, 1980s, 1990s, and 2000s, respectively. Acute glomerulonephritis (AGN), including post-*streptococcal* glomerulonephritis (PSGN), accounted for almost all of the PIGN cases in the 1970s, but decreased to approx. 40%–50% since the 1990s. In the 1990s, *Staphylococcus aureus* infection-related nephritis (SARN) showed a rapid increase in rate, reaching 30%. The incidence of hepatitis C virus infection-associated GN (HCVGN) has increased since the 1990s. The average age at onset rose from 33 to 51 years over the study period. These transitions can be summarized as increases in SARN and HCVGN and decreases in PSGN and other types of AGN, since SARN and HCVGN have older onsets compared to PSGN and other AGN types. The clinicopathological features were marked for each PIGN. Regarding the prognosis, the renal death rates of both the SARN and HCVGN groups were significantly higher than those of other PIGN.

**Conclusion:**

Based on our analysis of the Renal Biopsy Database, the incidence of PIGN in Japan reached its peak in the 1990s. The temporal changes in the incidence of PIGN reflected the trends in infectious diseases of each decade and the continual aging of the population, with a related higher susceptibility to infections.

## Introduction

Post-infectious kidney injuryhas several types of pathological manifestations, including glomerulonephritis, tubulointerstitial nephritis and acute tubular necrosis. Of them, post-infectious glomerulonephritis (PIGN) is associated with immunological response to infection and is mediated by various agents such as bacteria, viruses, fungi and parasites. The incidence of PIGN has decreased in the last 50 years in accord with the improvement of living environments and antibiotics [1].

The causes of PIGN have also changed with the times. In the past, most of the cases of PIGN were post-*streptococcal* glomerulonephritis (PSGN) in children with a prior infection of the throat or skin. Although the rate of PSGN cases has risen to 9.5–28.5 cases per 100,000 person-years in developing countries, the corresponding rate of developed countries continues to decline and is now estimated to be 0.3 cases per 100,000 person-years [2].

In recent years in developed countries, non-*streptococcal* infections—represented by *Staphylococcus aureus* or gram-negative bacterial infection in adults (especially in the elderly and in immunosuppressed patients)—have taken the place of *streptococcal* infection in children [1,3]. Human immunodeficiency virus (HIV) and hepatitis C virus (HCV) have also been far more widely recognized as causative agents of PIGN, even though they are not very common in Japan [4,5].

These transitions reflect the increase in many populations’ average life expectancy, accompanied by a constant rise in the geriatric population with a concomitant higher susceptibility to infections and higher prevalence of diabetes [3]. The epidemiology of PIGNs in several countries has been described [1], but few reports mention Asian countries including Japan [6]. The present study is the first report of the trend of PIGN cases in Japan from 1976 to 2009, predicated on a renal biopsy database.

## Patients and Methods

Based on 6,369 renal biopsies, including 143 necropsies, performed from 1976 to 2009 at the University of Tsukuba and eight local affiliated hospitals (in 2009, mainly in Ibaraki Prefecture, Kanto region, Japan), we calculated that approx. 200 biopsies per year were performed. All of the institutes obtained the patients’ written informed consent to undergo the renal biopsy before its performance. We analyzed the cases of the patients whose renal biopsy results demonstrated PIGN according to the following definitions, in reference to Heptinstall’s pathology of the kidney [7]:

PSGN (post-*streptococcal* glomerulonephritis): Acute glomerulonephritis (GN) after *streptococcal* infection as represented by pharyngitis or dermatitis with serological positivity (streptolysin-O) or hypocomplementemia, and histologically endocapillary proliferative GN at the active stage, mesangial proliferative GN at the healing stage or both types at a subclinical stage with C3-dominant depositions. Some large arch-like subepithelial electron-dense deposits (humps) on electron microscopy images are characteristic but not imperative for the definite diagnosis. Other type of AGN: Acute GN except for *streptococcal* infection or indeterminate *streptococcal* infection because the description of streptococcal infection was not provided on the medical record. SARN (*Staphylococcus aureus* infection-related nephritis): Various clinical and histological types of glomerular and tubular diseases occurred with *Staphylococcus aureus* infection, as represented by nephrotic syndrome and/or rapidly progressive nephritic syndrome and/or nephrotic syndrome in the clinical examination, and endo- and/or extracapillary proliferative GN with IgA-dominant immune complex depositions in histology [8]. HBVGN (hepatitis B virus infection-associated GN): Typically membranous or membranoproliferative GN occurred with hepatitis B virus infection. In practice, the characteristic histology for secondary membranous (such as mesangial hypercellularity, endocapillary proliferation, co-localized immunofluorescence of anti-IgA, IgM, C3, mesangial or sub-endothelial deposits shown by electron microscopy) or the membranoproliferative form with HBV infection is necessary. Anti-HBe antigen, IgG subclass and M-type phospholipase A2 stain are helpful for an accurate diagnosis, but not required for the definite diagnosis. HCVGN (hepatitis C virus infection-associated GN): Typically membranous or membranoproliferative GN occurred with hepatitis C virus infection. Mixed-type cryoglobulin or hypocomplementemia is occasionally positive, but is not required for the definite diagnosis. Other causes: PIGN except for the major diseases mentioned above, referred by the international World Health Organization (WHO) classification of glomerular diseases [9].

We excluded IgA-related GN including cirrhosis- or enteritis-related GN from the present analysis. Regarding the adaptation of renal biopsy for PIGN, especially PSGN, we performed renal biopsies from the 1970s through the 1990s in a proactive manner (thoughtful comments by Prof. Akio Koyama and Prof. Masaki Kobayashi). At the start of the 2000s, almost all of the practicing nephrologists in our group have managed to limit the adaptation to the atypical clinical form of PSGN. Meanwhile, the cases suspected SARN were candidates for renal biopsy at any time.

We recorded the demographic, clinical and pathological features and the renal outcomes of the PIGN patients at the times of their renal biopsies, in cooperation with the nephrologists involved in each case. To analyze the parameters, we used the chi-squared test or one-way analysis of variance (ANOVA) and the Tukey multiple comparison post-test. P-values <0.05 were considered significant. The cumulative survival was estimated with Kaplan-Meier method, and compared by log-rank test among each PIGN group.

We also indicated the changes in the rate of PIGN over four decades: the 1970s, 1980s, 1990s, and 2000s. However we didn’t compare the change in the rate with any statistics because we were unable to exclude the sampling bias based on the possibility to change the indication policy for renal biopsy during four decades. We examined the trend of the age changes of PIGN over the four decades by using the Jonckheere-Terpstra trend test. The IBM SPSS software package, ver. 22, was used for all of the statistical analyses. This research protocol was approved by the Ethics Committee of Tsukuba University Hospital (H26-175). Informed consent from each individual to participate in this study was not required by the Institutional Review Board, because the study was a retrospective review of clinical records and pathological results only. Instead of informed consent, an announcement of this study was posted on the University of Tsukuba and local affiliated hospitals.

## Results

### Characteristics of PIGN

Of the 6,369 total renal biopsies examined histologically from 1976 to 2009, 131 cases were diagnosed with PIGN (incidence rate 2.1%). All of the patients were Asian. The age of the PIGN patients ranged from 3 to 88 years, with an average age of 46±19 years. Ten children (less than 18 years old) were included. The male-to-female ratio of the PIGN cases was roughly 2:1, including 87 males and 44 females.

Forty-six cases of PSGN, 23 cases of other types of AGN (of a total of 69 cases of AGN), 26 cases of SARN, 13 cases of HBVGN, 20 cases of HCVGN, and 3 cases of other causes were included (Table 1). The age and gender data of each PIGN group were significantly different. The average ages of the PSGN, other AGN and HBVGN groups were younger than those of the SARN and HCVGN groups. The SARN, HBVGN and HCVGN groups were male-dominant.

**Table 1:** Clinicopathological findings of each type of PIGN.

	PSGN	Other AGN	SARN	HBVGN	HCVGN	p value
**number**	46	23	26	13	20	
**age (ave±SD yrs)**	36.9±18.1	38.4±15.0	60.7±14.8	33.8±11.4	57.8±10.6	p<0.01
**gender (number)**	27 / 19	8 / 15	24 / 2	10 / 3	15 / 5	p<0.01
**clinical syndrome (number)**	AGN 42, NS 2, CGN 2	AGN 22, NS 1	RPGN 8, NS7, RPGN+NS 6, CGN 3, CPH 2	CGN 8, NS 5	NS 9, CGN 9, RPGN 1, RPGN+NS 1	
**urinary protein (g/day)**	2.2±2.0	1.8±2.0	4.6±4.7	2.9±1.8	3.5±4.5	p = 0.05
**hematuria (%)**	95	100	90	50	63	p<0.01
**serum creatinine (ave±SD mg/dL)**	1.6±1.4	1.0±0.3	2.6±2.4	1.3±1.5	1.5±1.1	p<0.01
**hypertension (%)**	31	79	57	44	59	P = 0.02
**serum hypocomplement (%)**	80	87	20	46	59	p<0.01
**necropsy (%)**	0	0	31	15	0	p<0.01
**renal transplantation (%)**	0	0	0	0	5	p = 0.24
**histology**	EndoPGN 40, MesPGN 6	EndoPGN 21, MesPGN 2	MesPGN 12, MPGN 6, MesEndoPGN 4, EndoPGN 4	MPGN 6, MN 6, FGS 1	MPGN 13, MN 5, MesPGN 1, ITG 1	
**comcomitant state (%)**						
** malignancy**	0	0	20	8	5	p = 0.01
** diabetes**	7	5	16	0	11	p = 0.49
** operation**	0	0	40	0	5	p<0.01
** HCV infection**	0	0	17	0	100	p = 0.04*
** liver cirrhosis**	0	0	8	8	21	p = 0.02

*Comparison of the four PIGN types other than HCVGN. PSGN, post-*streptococcal* glomerulonephritis; other AGN, acute GN except for *Streptococcal* infection or undetermined of *Streptococcal* infection; SARN, *Staphylococcus aureus* infection-related nephritis; HBVGN, hepatitis B virus infection-associated GN; HCVGN, hepatitis C virus infection-associated GN; ave, average; SD, standard variation; NS, nephrotic syndrome; CGN, chronic GN; RPGN, rapidly progressive GN; CPH, chance proteinuria and hematuria; EndoPGN, endocapillary proliferative GN; MesPGN, mesangial proliferative GN; MPGN, membranoproliferative GN; MN, membranous nephropathy; FGS, focal segmental glomerulosclerosis; ITG, immunotactoid glomerulopathy.

The clinical renal syndrome was expectedly characteristic for each PIGN group; e.g., acute glomerulonephritis syndrome in PSGN, and rapidly progressive glomerulonephritis or nephrotic syndrome in SARN. Among the clinical parameters, the rates of hematuria, serum creatinine value, existence of hypertension and hypocomplementemia differed significantly among the PIGN groups, but the urinary protein level did not. Approximately 30% of the SARN cases were performed by necropsy. The renal histology was expectedly characteristic for each PIGN, such as endocapillary proliferative glomerulonephritis in PSGN and AGN, and various features of proliferative glomerulonephritis in SARN. The cases of the patients with SARN tended to be complicated by malignancy and/or surgery, and the HCVGN cases often included liver cirrhosis.

**Fig 1:**
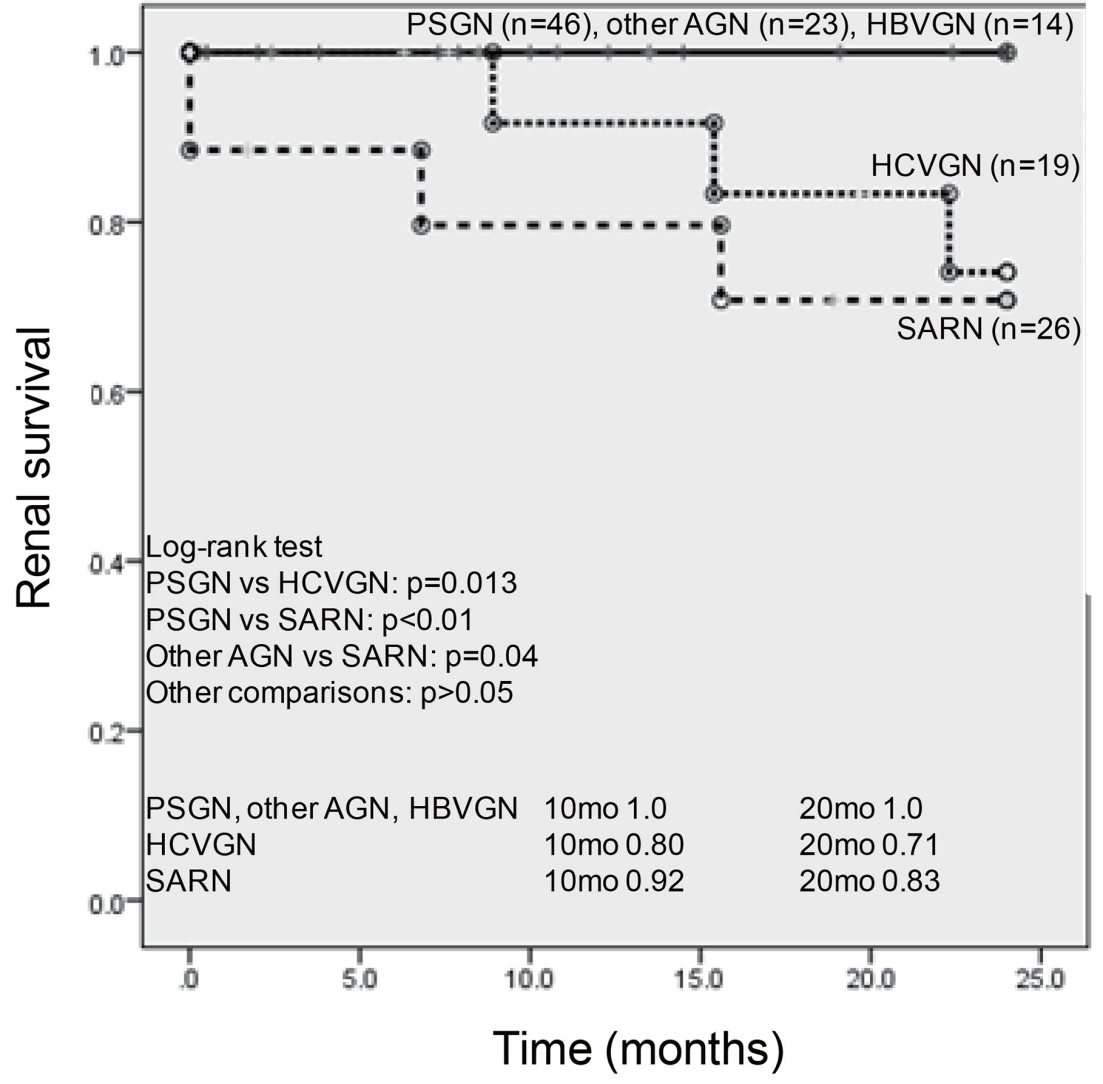
The comparison of renal survival rates in each PIGN group. PSGN, post-*streptococcal* glomerulonephritis; other AGN, acute GN except for *streptococcal* infection or undetermined of *streptococcal* infection; SARN, *staphylococcus aureus* infection-related nephritis; HBVGN, hepatitis B virus infection-associated GN; HCVGN, hepatitis C virus infection-associated GN. n.s.: not significant, mo: months.

The renal outcomes of each PIGN group are summarized in Fig 1. In a 24-month follow-up period, no patient with PSGN, other AGN or HBVGN reached end-stage kidney disease. In contrast, the renal death rates of both the SARN and HCVGN groups were significantly higher than those of other PIGN. Regarding the survival rate of each PIGN group, the tendency for lower survival in the SARN group was similar to the higher rate of necropsy upon its diagnosis (data not shown).

The three cases of ‘other’ causes of PIGN were one case of syphilis-associated membranous nephropathy (in 2006), one case of human immunodeficiency virus-associated immune-complex type GN (in 2008), and one case of endocapillary proliferative GN after pneumonia caused by an unknown bacterium (in 2009). No tropical infections such as malaria or dengue fever were observed.

### Temporal changes in the rate of PIGN

We indicated the rates of PIGN over four decades (the 1970s, 1980s, 1990s, and 2000s). The rate of PIGN was 2.4% (8/328) in the 1970s, 1.1% (18/1,631) in the 1980s, 2.6% (65/2,464) in the 1990s, and 2.1% (40/1,946) in the 2000s. Thus the rate of PIGN fell from the 1970s to the 1980s and then increased from the 1980s to the 2000s.

**Fig 2:**
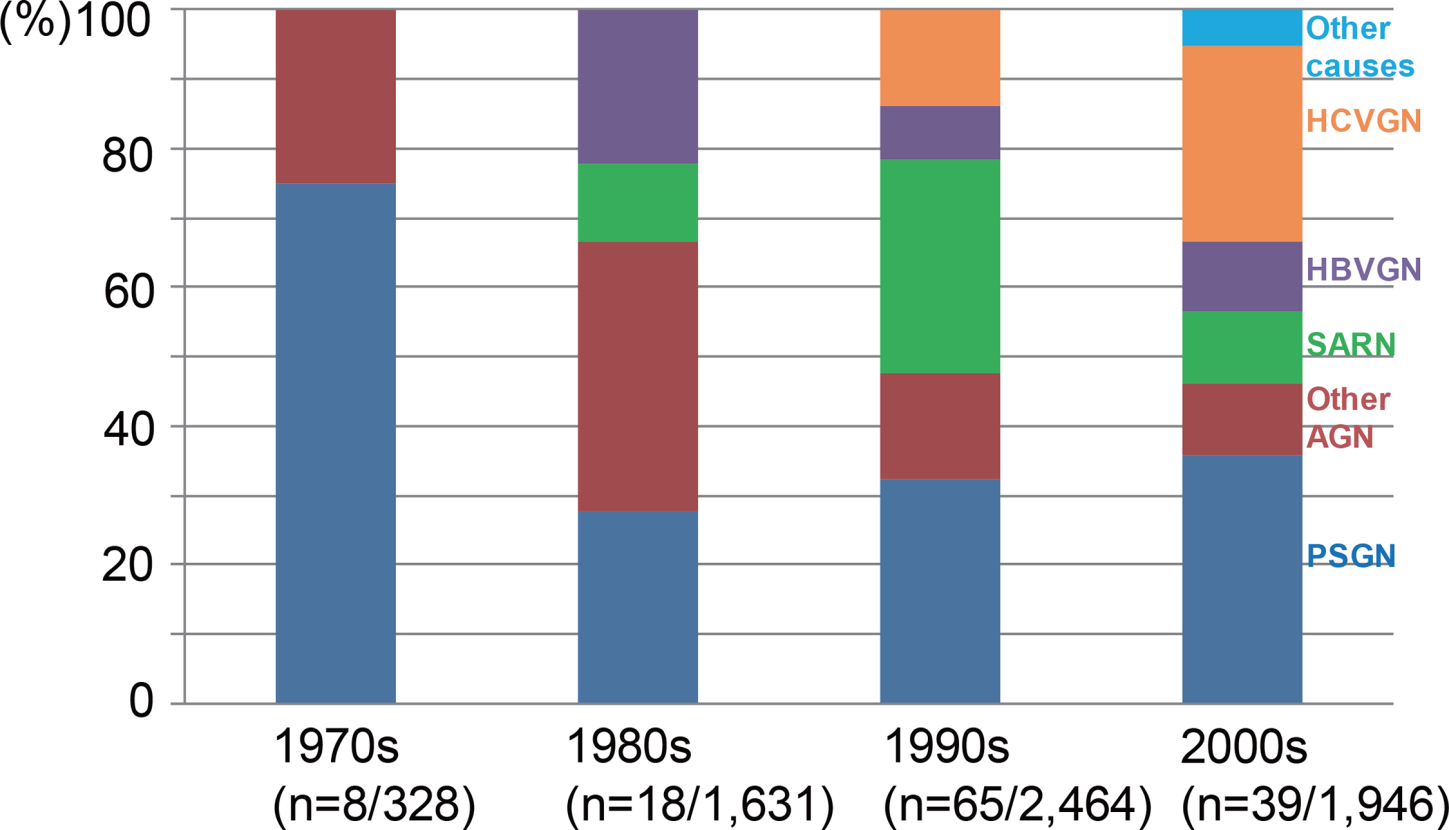
The temporal changes in the detailed contents in PIGN over the four-decade observation period. Abbreviations are explained in the Fig 1 legend.

We next examined the temporal changes in the sub-categories of the PIGN cases over the four decades studied (Fig 2). The prevalence of AGN (including those of both PSGN and other AGN) accounted for almost all of the PIGN cases in the 1970s, decreasing to 40%–50% and remaining at that level after the 1990s. PSGN accounted for more than 70% of the cases until the 1970s, falling to approx. 30% and remaining at that level from the 1980s onwards. By contrast, in the 1990s, the rate of SARN cases showed a rapid increase, rising to 30%, and HCVGN cases accounted for approx. 30% of the PIGN cases in the 2000s.

A patient who had been diagnosed as having hepatitis without hepatitis A virus (HAV) or HBV appeared with MPGN in the 1990s, and he then turned up in the records with an HCV infection and HCVGN. No sample of GN associated with hepatitis without HAV or HBV was collected during the 1970s and 1980s. The prevalence of HBVGN varied during the observation.

### Temporal changes in the age and gender of the PIGN patients

**Fig 3:**
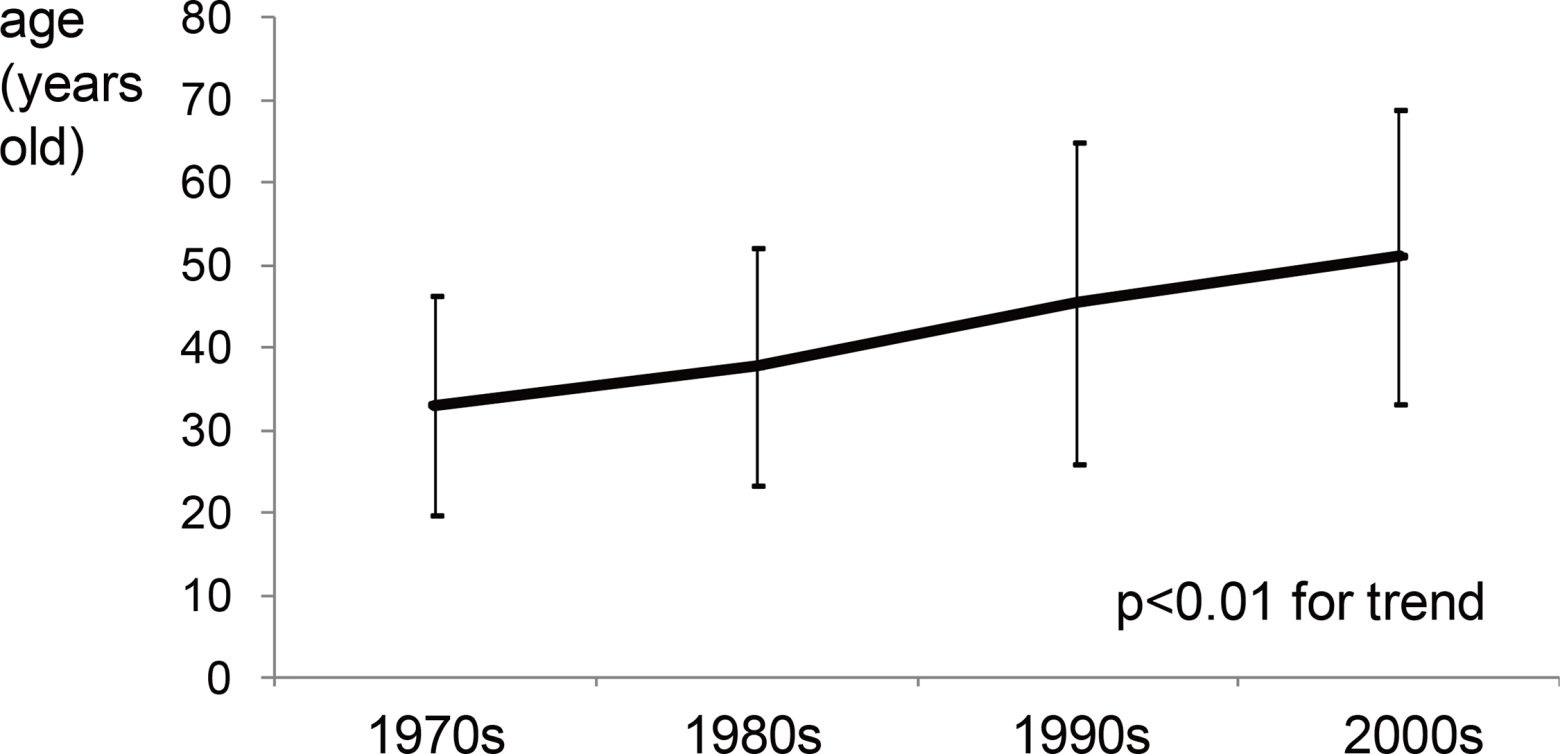
The temporal changes in the age of the onset of PIGN over the four decades. Average age ± standard variation.

We then examined the temporal changes in the age of the PIGN patients from the 1970s to the 2000s. The age at which the patients presented continued to increase from the 1970s to the 2000s [Fig 3, 33±13 yrs in the 1970s, 38±14 yrs in the 1980s, 45±19 yrs in the 1990s and 51±18 yrs in the 2000s, by the Jonckheere-Terpstra trend test (p <0.01 for trend)]. Moreover, the average age of each PIGN group including the PSGN group did not change significantly over several decades (Table 2). Thus, with reference to the temporal changes in the prevalence of PSGN, other AGN, SARN and HCVGN cases, the increases in the prevalence of SARN and HCVGN cases and a decrease in the prevalence of PSGN and other AGN cases influenced this aging transition.

**Table 2:** The temporal change of the age in each PIGN over the four decades.

	1970s	1980s	1990s	2000s	p value
**PSGN**	33±15	39±26	34±18	42±19	p = 0.59
**Other AGN**		40±12	33±11	50±23	p = 0.16
**SARN**			62±13	65±13	p = 0.77
**HBVGN**		35±8	30±18	37±6	p = 0.68
**HCVGN**			54±9	61±11	p = 0.13

The average age ± SD in each group was calculated. A blank indicates that <3 patients were available. Abbreviations are explained in the Table 1 footnote.

We also investigated the temporal changes in the male-to-female ratio of the PIGN case over the 40-year study period. The male-to-female ratio of the PIGN cases, roughly 2:1, was similar through the 1970s, 1990s and 2000s (data not shown).

## Discussion

The causes and the frequency of the cases of PIGN in Japan have changed from the 1970s to the 2000s. We also reviewed the literature to examine the corresponding situation in other countries, and we compared the trends of other developed countries with those of Japan.

The trends of PIGN in other developed countries have been reported, including the U.S., France and Taiwan [6,10,11], and in those countries too, the microbial agent associated with PIGN has shifted from *Streptococcus* to *Staphylococcus aureus*. In 2011, Nasr et al. reported the prevalence of PIGN in patients over 65 years old in the U.S. from 2000 to 2010, and the causative agent was *Staphylococcus* in 46% and *Streptococcus* in 16% [10]. Wen et al. reported that the causative agent was *Staphylococcus* in 57.1% of the PIGN cases in Taiwan from 2000 to 2009 [6]. Not only the percentage of PSGN cases in the total number of PIGN cases but also the number of PSGN cases themselves has continued to decrease according to the several reports [12,13].

Simon et al. reported that the number of PSGN cases was significantly decreased between 1976 and 1990 in a French region [9], and Roy et al. described a decrease in PSGN cases in children from 310 cases in the 1960s to 95 cases in the 1980s [10]. As in Japan, these results indicated the decrease of the rate of each PIGN including PSGN. Additionally, Takeda et al. reported that in Japan both the number of child patients with streptococcal infection and the number of PSGN cases decreased from 1975 to 2005 [14]. Taking all of the above-mentioned findings as a whole, it appears that both the rate and the numbers of PSGN have recently decreased in the developed countries, including Japan.

The temporal changes in PIGN may also be reflections of the trends in infectious diseases during each decade and the continual aging of the population, with higher susceptibility to infections. Actually, the numbers of cases with AGN including PSGN seem to be decreasing in recent years. However, the increases in the numbers of SARN and HCVGN cases are more important in the developed countries.

The percentage of adult PIGN patients increased from 4%–6% to 34% over the past 40 years, compared to child PIGN cases [1]. It is possible that the aging of the population that is occurring in almost all developed countries has influenced the onset of some infections, including *Staphylococcal* infection. Additionally, co-morbid conditions such as malignancy, diabetes, post-surgical complications and liver cirrhosis are more common in elderly persons. These co-morbid conditions are associated with *Staphylococcal* infection and were seen in two-thirds of the cases examined in a previous study, compared to only 8% associated with *Streptococcal* infection [3]. SARN is thus expected to becomes to be a major GN among the PIGNs, and patients with PIGN of all types are now rapidly aging.

Viral infection is also considered a significant factor in PIGN. Chronic HCV infection has affected an estimated 170 to 200 million people worldwide. In the U.S., 4.1 million people have antibodies to HCV and 3.2 million are chronically infected [4]. In 1993 (4 years after the identification of HCV in 1989), an association between MPGN and HCV was reported. Although the incidence of HCVGN is unknown due to the lack of large-scale studies [15], Arase et al. reported 188 autopsy cases with HCV infection, with glomerular lesions in 54.8% of the cases [16].

In another study of renal biopsies from 30 liver transplant patients with HCV-associated cirrhosis, 25 patients had immune complex glomerulonephritis, 12 of which were MPGN, followed by seven cases of IgA nephropathy and six of mesangial proliferative glomerulonephritis [17]. El-serag et al. noted that cryoglobulinemia was more common in HCV-infected individuals than controls (0.57% vs. 0.05%), and HCV-infected individuals had a greater prevalence of MPGN (0.36% vs. 0.05%) [18].

Worldwide, approx. 4.9 million people are newly infected with HIV each year, and in the U.S., there are 1 million HIV-infected individuals with approx. 40,000 people newly infected each year [4]. HIV infection can cause many types of kidney diseases, such as HIV-associated nephropathy (HIVAN), immune complex-type GN, and thrombotic microangiopathy. HIVAN was first reported in 1984 (3 years after the first identification of HIV in 1981), and it is the most frequent renal manifestation among HIV-associated kidney diseases.

According to a report in the U.S., 93% of the renal biopsies of 112 HIV-positive patients showed glomerular lesions, manifesting focal segmental sclerosis patterns in most cases [4]. HIV-positive patients are frequently positive for HCV as well. In developing countries, not only HCV or HIV but also schistosomiasis and malaria have been reported as the cause of renal diseases [19].

A point in common between Japan and other countries is that HCVGN has been considered a burden. In the U.S., the burden of HCV has been increasing along with the aging of HCV-infected patients, while the number of newly infected patients decreased after 1989 [20]. Likewise, in Japan, the prevalence of HCVGN has gradually increased since the 1990s with an increasing presence among PIGN cases. On the other hand, contrary to the high prevalence of HIV-associated kidney disease in the U.S., there was only one case with HIV-associated kidney disease in our Renal Biopsy Database.

The present report is the first to describe PIGN trends in Japan over four decades and to compare the trends with those of other developed countries. The PIGN trends in Japan will continue to change along with the aging of the population and the increase in diseases such as diabetes and HIV infection. We hope that this first report from Japan on PIGN will provide a comparative perspective on the changing epidemiology of this condition.
